# Searching for Judgment Biases Among Elite Basketball Referees

**DOI:** 10.3389/fpsyg.2018.02637

**Published:** 2018-12-20

**Authors:** Elia Morgulev, Ofer H. Azar, Ronnie Lidor, Eran Sabag, Michael Bar-Eli

**Affiliations:** ^1^The Academic College at Wingate, Wingate Institute, Netanya, Israel; ^2^Kaye Academic College of Education, Beer-Sheva, Israel; ^3^Department of Business Administration, Guilford Glazer Faculty of Business and Management, Ben-Gurion University of the Negev, Beer-Sheva, Israel; ^4^Laboratory of Economic Behavior of the Center of Psycho-Economic Research, Povolzhsky Institute of Management named after P.A. Stolypin, RANEPA, Saratov, Russia

**Keywords:** biases, judgment, decision making, sport referees, basketball

## Abstract

An attacking basketball player initiating significant physical contact with a defender who has already established a legal and stationary position, should be called with an offensive foul. Offensive foul situations are particularly ambiguous and complex, making the referee’s task a difficult one. In such conditions of complexity and constraints of time, the referee is likely to be prone to systematic biases, as has been documented by previous research in other sport settings. We analyzed the referees’ decisions in 250 instances of collisions between an attacking player and a defender. In these collisions the defender fell, and potentially an offensive foul could be called. We found no evidence of favoritism granted to the home team, to star players, or to high-reputation teams, or of small players being tackled by significantly larger opponents. The findings suggest that these biases are not very robust, and are sensitive to the context, and that proper training of referees and enhanced awareness can help to alleviate referees’ biases.

## Introduction

Highly qualified managers and employees are often required to make important decisions under severe time and information constraints. This is especially true in high-profile jobs such as surgeons, brokers, pilots, and sport referees (Dawson, 2012). Professional sport referees can be thought of as highly qualified agents, authorized to act on the court on behalf of stakeholders (principals) such as FIFA, the NBA, the IOC, etc. These agents are expected to act in the best interest of their principals. Consequently, a basic requirement for sport referees is to judge impartially, since unbiased referee judgment is necessary for the accomplishment of the principal’s objective—namely, to secure the integrity and legitimacy of any professional sport competition.

Agency theory literature typically emphasizes the role financial incentives play on agents, causing them to depart from honest behavior (e.g., bribes, promotions) (Garicano et al., 2005). This implies that the principal must align the referee’s interests with his/her own in order to secure the referee’s impartiality. Yet, Brand et al. (2006) argued that as long as a substantial gray area exists between legal and illegal contacts (e.g., fouls) in most sports, the interpretation of the contact by the referee cannot be based solely on the written rule system (i.e., formal cues). In this context, sociologists and social psychologists suggest that agents’ decisions are not solely determined by direct financial rewards, but are also driven by non-material payoffs that emerge in the agents’ social environment, in the form of social approval or social sanctions (Dohmen and Sauermann, 2016). Dawson (2012) stated that in circumstances of time and information constraints, social pressure can lead to systematic biases in decision making, since individuals rely on heuristics and emotion in framing their decisions. In this regard, Garicano et al. (2005) indicated that aside from financial incentives, the role of social forces in corrupting the behavior of individuals needed to be further examined. In this study, we turned to basketball to investigate referees’ decisions in offensive foul situations, to determine whether basketball referees are prone to various biases that have been reported by previous literature in other sport settings.

Dohmen and Sauermann (2016) pointed out that referees in professional sports are a perfect object for empirical investigation of the existence of biases and their underlying social forces: First, referees’ decisions are observable and publicly available for analysis; Second, professional referees are paid to be accurate and impartial, and their performance is monitored and evaluated.

A growing body of literature has addressed decision making processes and biases of sport referees. We will focus here on findings that are relevant to the aims of our study.

### Home Team Favoritism Bias

Nevill et al. (2002) used a laboratory setting to examine the influence of noise generated by a crowd on officials’ decisions. In their study, referees watched various challenges recorded on videotape under sound vs. no-sound conditions. It was found that the presence of crowd noise had a significant effect on the decisions made by the referees. A similar approach was adopted by Unkelbach and Memmert (2010) who used video clips from 56 different matches from the German Bundesliga. These researchers demonstrated that observers were more likely to award yellow cards under noisy conditions.

Relying on archived data, Sutter and Kocher (2004) examined referees’ decisions in awarding penalties and extra time at the end of football matches of the German Bundesliga, and reported on the existence of home bias. That is, football referees were much more likely to award penalties to the home team than to the visiting team. Referees were also found to add significantly more extra time in cases where the home team is behind by one goal than when it is ahead by one goal, or when there is a draw after 90 min of play. Garicano et al. (2005) extended the finding on biased extra time allocation to behavior of Spain’s top-league referees. Dawson and Dobson (2010) analyzed data from five seasons of European cup football matches and found that referees favor home teams when awarding yellow and red cards. Page and Page (2010) used a dataset which contains information from 37,830 individual matches; they concluded that the home advantage effect differs significantly among referees, and that this relationship is moderated by the size of the crowd.

In other sports, Balmer et al. (2005) reported that judges enhance home advantage in European championship boxing. Crowd and noise were found to increase the scores of Muay Thai judges, resulting in an advantage to the home competitor (Myers et al., 2012). In basketball, it was reported by Anderson and Pierce (2009)—based on 365 NCAA games—that there is a significant bias toward officials calling more fouls on the visiting team. Price et al. (2012) analyzed play-by-play data for all NBA regular season and playoff games from the 2002–2003 through 2007–2008 seasons, and reported that referees favor the home teams in their calls. Yet, compared to football, the research on home favoritism in basketball is scarce and inconclusive.

Beginning March 2015, the NBA began to release play-by-play reports regarding all calls in close game situations with two or less minutes to play. Deutscher (2015) used these official reports to analyze discrepancies between actual calls by the referees and the judgment of these foul situations by the principal—the League. Analysis of 1,229 referees’ calls from 113 close games found no support for home team favoritism in the NBA. Deutscher stressed that such a comparative analysis has an advantage compared to previous research that relied on analysis of statistical frequency of calls, identified patterns, and interpreted these patterns in terms of biased decision making. This had the potential result of mixing biased decision making with actual differences in the behavior of players and teams.

### Star-Player Favoritism and “Big Team” Favoritism Bias

Lehman and Reifman (1987) demonstrated that star players in the NBA had fewer fouls called (against them) at home than away, whereas non-stars were not subject to this pattern. The authors concluded that player status creates additional pressure from the crowd on the referee when the “star” is playing at home. Findlay and Ste-Marie (2004) found that a reputation bias does exist when judging figure skating, and that it is present during the evaluation phase of sport performance appraisal. Mills (2014) reported on favoritism of referees toward players with a higher status in Major League Baseball. Caudill et al. (2014) suggested that referees are “protecting” the league’s star players, as they found that NBA All-Star players were awarded with an additional 0.32 free throw attempts per minute during the fourth quarter of NBA Playoff games, the most critical games of any season for players, coaches, owners, and fans.

On the team level, Lago-Peñas and Gómez-López (2016) showed that football referees favor big teams (i.e., more famous and higher-ranked teams) by shortening close games when the big team is ahead and extending close games when the big team is behind.

### Player’s Size Bias

Van Quaquebeke and Giessner (2010) reported that height is one of the decision cues used by referees when judging tackles in football (soccer), despite the fact that body composition is not part of the formal foul-related information that referees should take into consideration. The authors found that taller players are likely to be perceived as more aggressive; thus, they are identified as foul perpetrators and their respectively smaller opponents as foul victims. We speculate that in the situation of an offensive foul in basketball, a collision initiated by a much bigger player (who may be seen as more aggressive) may incline the referee in favor of the smaller player. In this regard, prior information about players’ aggressiveness was found to influence referees’ decisions in football (Jones et al., 2002; Strauss and Pier, 2002).

To this end, the literature contains a wealth of articles documenting home team favoritism in football. However, home team favoritism in basketball has remained less studied, and the emerging picture is ambiguous. For instance, a comparative analysis of basketball calls performed by Deutscher (2015) elicited no evidence for home bias or favoritism toward superstar players. In the NBA, Gift and Rodenberg (2014) analyzed referees’ decisions in 4,463 regular season games from 2008 to 2012, and reported that more personal fouls were called when a relatively shorter referee officiated the game. Yet, no evidence was found that the rate of foul calling varies with the players’ height. Additionally, Pope et al. (2018) reported that awareness of biases can mitigate their effects. Their analysis revealed that the widespread media attention highlighting racial bias among professional basketball referees led to the complete disappearance of the bias.

We aim in this study to implement a setting of an offensive foul in order to determine if biases that were discussed in the literature, mainly in football, also hold for referees in the game of basketball. Morgulev et al. (2014) used the setting of an offensive foul in basketball for assessing referees’ and players’ decision making from a cost-benefit perspective. In their study, 501 collisions between attacking players and defenders were analyzed by participating experts (elite-level basketball referees). The experts identified 65% of the falls in the sample as voluntary (flops), meaning that the defenders deceived by deliberately falling in order to manipulate the referee.

This implies that the setting of an offensive foul is a challenging one for a referee. In such an ambiguous setting, the referee may succumb to non-formal but easily accessible cues. Size of the player appears to be a potential cue since the referee may be inclined to identify the smaller player as a foul victim. Favoritism of star players was proposed by the literature as a potential bias in basketball refereeing. Analogically, appeasing the home crowd also seems as the natural thing to do when the situation is uncertain. On top of it, there are available data on players’ sizes and positions, teams’ ranks, points scored, and minutes played, enabling to construct the above-mentioned variables for analysis. We hypothesize that in offensive foul situations, referees are inclined in favor of smaller players, players performing in front of their home crowd, players playing for bigger teams, or players with higher reputation.

## Materials and Methods

The current research meets the requirements for a waiver from ethical approval, since it is based on information freely available in the public domain.

### Materials

A sample of offensive foul situations previously analyzed by Morgulev et al. (2014) was reviewed, and additional variables were collected. Morgulev et al.’s sample consisted of incidents between an attacking player and a defender that were identified by professional basketball coaches as incidents that had the potential to meet the criteria of an offensive foul (an attacking player with the ball pushing or moving into an opponent’s torso while the defender had already established a stationary position): 250 incidents where the defender fell after contact with the offensive player and 251 incidents where the defender remained standing after the contact.

These incidents were taken from games played in the 2009/10 season of the Israeli Basketball Super League. It should be pointed out that Israeli basketball is at a high international level. For example, Maccabi Tel Aviv has won the FIBA European Champions Cup several times, and one of its recent coaches, David Blatt, went on to be the head coach of the Cleveland Cavaliers, which he led to the 2015 NBA Finals. In the current study, we focused on the 250 cases where the defender fell.

### Procedure

We reviewed these 250 incidents and documented whether the referee called an offensive foul, and whether the attacking team was the home or away (guest) team. For each player we recorded three performance parameters from the official site of the Israel Basketball Super League: (1) annual average scoring per game; (2) minutes played per game; (3) overall “performance index rating” per game. An individual “performance index rating” is calculated by the addition and subtraction of positive and negative player’s actions (e.g., points made, rebounds, fouls drawn, missed field goals, fouls committed, etc.). Performance index rating is an official metric used to rate players in the “EuroLeague,” as well as in various European national leagues.

Incidents with at least a 10-unit difference between the attacking and defending players in all three of the parameters were denoted as “attacking player favorite” or “defending player favorite.” Namely, a defender who averaged at least 10 points more per game, played at least 10 min more per game, and had a performance index rating at least 10 units higher than the attacking player, was denoted as the favorite in the given incident.

Ranks of the teams by the end of the 2009/10 season and the end of the preceding season were used as a proxy for team status. Four teams stood out in both seasons and therefore were assigned with “big team” status. Games between one of the big teams and any other team in the league were denoted as “attacking team favorite” or “defending team favorite” (the favorite being the big team).

Additionally, we recorded the player’s position (e.g., small forward, power forward, shooting guard, etc.) and the height of both players in each incident—parameters that were used to evaluate the body size of the players. In cases of incidents between players playing in fundamentally different positions (e.g., point guard vs. power forward, shooting guard vs. center, etc.) combined with at least a 10-cm height difference between the players, the cases were denoted as “smaller attacking player” or “smaller defending player.” A summary of the four variables constructed for the analysis is presented in Table 1.

**Table 1:** Variables used to test for officiating biases in offensive foul situations.

	Criterion	Criterion	Criterion
Home advantage	Defender playing at home		
Player reputation advantage	Defender scored at least 10 more points per game	Defender played at least 10 more minutes per game	Defender had at least 10 more positive actions per game
Team reputation advantage	Four teams achieved the highest ranks in 2008/9 and 2009/10 seasons		
Body size advantage	Point guard or shooting guard defending against power forward or center	Defender at least 10 cm shorter	


### Data Analysis

Chi-square analyses were performed in order to indicate the degree of the relationships between the referees’ calls and home team favoritism, players with higher reputation favoritism, bigger teams favoritism, and smaller defenders favoritism. As the response variable in our study was binary (i.e., has two possible outcomes: foul vs. no foul), the assumptions necessary to conduct ANOVA were likely to be violated. Nevill et al. (2002) indicated that logistic regression is a more appropriate technique in such cases. This analysis will estimate the odds associated with the two outcomes and how these will vary due to differences in the independent variables. Consequently, to verify the robustness of our results, we used a binary logistic regression model (Logit) and assessed the explanatory power of the four mentioned variables on referee’s decision to call an offensive foul.

## Results

### Home Bias

As mentioned in the introduction, studies in various sports found a home bias, according to which the referee gives the home team favorable treatment (e.g., Sutter and Kocher, 2004). Because our sample consists of ambiguous situations in which it is not clear if an offensive foul should be called or not, these situations are relevant for the home bias to appear. Table 2 presents the frequency of calls for an offensive foul for home vs. away teams. We can see an almost equal distribution of offensive fouls awarded for defenders of the home and the guest teams. A statistical test confirms that our data show no support for the home bias documented in other studies: χ^2^(1, *N* = 250) = 0.015, *p* = 0.902.

**Table 2:** Frequency of calls for an offensive foul for home and away teams.

	No call for offensive foul		Call for offensive foul		Total
Away	99	80.5%	24	19.5%	123
Home	103	81.1%	24	18.9%	127
Total	202	80.8%	48	19.2%	250


### Players’ Reputation Differences

As mentioned earlier, the literature includes evidence for favorable treatment that “stars” receive from referees (e.g., Lehman and Reifman, 1987). We described above the three criteria we used to measure reputation, and we required all three to have a large difference (over 10 units) between the two players in order to categorize the case as having reputation differences. Therefore, a reasonable conjecture based on the literature is that the higher-reputation player (when such a large reputation difference exists) will enjoy favorable treatment from the referee (i.e., fewer fouls will be called against him/her and more fouls will be called in his/her favor). Table 3 presents the distribution of calls for offensive fouls by the difference in players’ reputation.

**Table 3:** Frequency of calls for an offensive foul by the players’ reputation differences.

	No call for offensive foul		Call for offensive foul		Total
Attacking player favorite	33	84.6%	6	15.4%	39
Similar reputation	157	80.1%	39	19.9%	196
Defending player favorite	12	80.0%	3	20.0%	15
Total	202	80.8%	48	19.2%	250


We can see in Table 3 that the frequency of calls enjoyed by “stars” in our study is not much different from the frequency of calls awarded to their less renowned fellow players. Statistical analysis supports the conclusion that there are no significant differences in the chances to get a call for offensive foul based on reputation. When we analyze the three categories in Table 3 we obtain χ^2^(2, *N* = 250) = 0.434, *p* = 0.805. When we exclude the “similar reputation” category and compare only the cases where one player enjoys a higher reputation, we obtain χ^2^(1, *N* = 54) = 0.166, *p* = 0.684.

### Teams’ Reputation Differences

Favoritism of “bigger” (more famous, higher-ranked, etc.) clubs by referees is another bias that was documented in the literature (e.g., Lago-Peñas and Gómez-López, 2016). Using the league rankings in the 2008/09 and 2009/10 seasons, we identified four clubs that clearly stood above the rest in the Israeli Super League. If referees favor bigger teams as reported in the literature, it should imply in our context that big clubs will enjoy more calls for an offensive foul against the opposite team. Table 4 presents the distribution of calls for offensive fouls, divided by the teams’ reputation difference.

**Table 4:** Frequency of calls for an offensive foul by the teams’ reputation differences.

	No call for offensive foul		Call for offensive foul		Total
Attacking team favorite	68	81.0%	16	19.0%	84
Similar reputation	85	81.7%	19	18.3%	104
Defending team favorite	49	79.0%	13	21.0%	62
Total	202	80.8%	48	19.2%	250


Table 4 suggests that referees did not call more fouls in favor of the “big teams,” χ^2^(2, *N* = 250) = 0.184, *p* = 0.912. When we exclude the “similar reputation” category and compare only the cases where one team is more of a favorite, we obtain χ^2^(1, *N* = 146) = 0.083, *p* = 0.774. It is worth mentioning that “big teams” are usually loaded with big names. As a result, players from such teams will often expose the referee to the aggregated effect of a star player playing for a high-caliber team. Yet, similar to the case of the players’ reputation difference, as in the case of difference in teams’ reputations, we did not observe any referee bias despite some of the literature showing such bias in other cases.

### Body Composition Differences

Van Quaquebeke and Giessner (2010) showed that referees tend to treat physically larger players as foul perpetrators, whereas their respectively smaller opponents tend to be viewed as foul victims. As explained in detail earlier, based on the players’ positions and players’ height we were able to identify collisions where the defender was significantly smaller. Therefore, according to the associated refereeing bias suggested in the literature, in these cases the defender was expected to receive favorable treatment from the referee. Table 5 presents the analysis of calls for an offensive foul, divided by body composition differences.

**Table 5:** Frequency of calls for an offensive foul by body composition differences.

	No call for offensive foul		Call for offensive foul		Total
Smaller attacking player	18	81.8%	4	18.2%	22
Similar size	164	82.0%	36	18.0%	200
Smaller defending player	20	71.4%	8	28.6%	28
Total	202	80.8%	48	19.2%	250


Table 5 shows that body composition, as opposed to the factors analyzed above, yielded some evidence for a bias as suggested in the literature. We can see a tendency in the referees to award smaller defenders more calls for an offensive foul in their favor (28.6 vs. 18.0% with similar size and 18.2% with a smaller attacking player). However, this effect is not statistically significant: χ^2^(2, *N* = 250) = 1.785, *p* = 0.410. When we exclude the “similar size” category and compare only the cases where one player is bigger, we obtain χ^2^(1, *N* = 50) = 0.729, *p* = 0.393. It is interesting to note that there is no meaningful difference in the frequency of offensive fouls between the cases of a smaller attacking player and cases of similar size players.

All the four variables that were analyzed above were reported in the literature as factors that may cause referee bias in sport, but in our data we find no evidence for such bias, especially in the cases of the home bias, or the reputation of the player or the team.

As a check of the robustness of our results, we analyze below the simultaneous effects of these four factors, using a logistic regression in which the dependent binary variable is whether the referee called an offensive foul, and the explanatory variables are those which we explored above. Table 6 presents the results of this regression.

**Table 6:** Logistic regression explaining the referee’s decision whether to call an offensive foul.

Variable	Coefficients		*p*-value
	B	SE	
Defender is playing at home	-0.018	0.163	0.912
***Player’s reputation difference***			
(reference category: defender’s reputation is lower)			
Similar reputation	0.325	0.492	0.509
Defender’s reputation is higher	0.380	0.817	0.642
***Team’s reputation difference***			
(reference category: defender’s team reputation) is lower)			
Similar reputation	-0.114	0.385	0.767
Defender’s team reputation is higher	0.053	0.437	0.903
***Body composition difference***			
(reference category: defender’s size is bigger)			
Similar size	0.017	0.586	0.977
Defender’s size is smaller	0.636	0.698	0.362


The results presented in Table 6 confirm our previous analyses and suggest that home advantage, the players and team’s reputation, and body size do not affect the referee’s decision whether or not to award an offensive foul.

## Discussion and Conclusion

The situation of an offensive foul in basketball involves complex and uncertain circumstances, where a decision should be made very quickly. Such a setting should provide fertile ground for refereeing biases to take place. In this study, we examine whether biases documented in previous literature (mainly in football) will also take place in the setting of an offensive foul in basketball.

We analyzed referees’ calls in 250 such incidents, taken from an entire season of the Israeli Basketball Super League, and we found no evidence for favoritism. That is to say, although the situation of potential offensive fouls should be prone to refereeing biases, in our sample the reputation of the players and the teams, the issue of home vs. away teams, and the players’ physical size did not significantly affect the frequency of calls for an offensive foul. The data provides some limitations to be mentioned. First, there is information only on decisions in offensive foul situations. Referee bias could prevail in a multitude of other calls made by a basketball referee. Second, we focused on relatively rare instances of offensive foul situations that had to be identified as such by experts, and we ended up with a relatively small sample of 250 cases—this after scanning through an entire season of games. The rarity of offensive fouls in basketball also made it challenging for us to look for sequential effects in referees’ decisions, phenomenon known as game management. We believe that by using recent technological advances, a larger sample can be examined.

The current null results are in line with those of Deutscher (2015), who found no evidence that referees’ decisions on fouls in the NBA are influenced by a player’s reputation or succumb to home favoritism. Additionally, Gift (2017) used detailed fighter performance statistics to investigate round-by-round judging decisions for major mixed martial arts (MMA) events. His findings have not supported the claim that judges favor previous titleholders. All this suggests that the refereeing biases previously reported in the literature are not so ubiquitous as hitherto believed.

The publication bias that has existed for a long time in many fields has resulted in papers with null results not being published, and a literature containing studies with many results that have later been found to be non-replicable. The unwillingness of referees and editors for many years to publish null results led researchers to avoid attempts to replicate studies, and to shun writing and submitting papers with null results. Even when they did, these papers were usually not published in outlets that are likely to get much attention. The resulting publication bias has led to erroneous literature, where many effects were believed even though they were not real, because the results were reported when an effect was found but not when an effect was not found. The recent understanding of the danger that this type of publication bias creates has led to attempts to make scholars more aware of this issue. Consistent with this approach, our study attempted to extend prior results to a new context, and subsequently no support was found for these results.

A possible contributing factor to our findings that do not reveal refereeing biases is the training that the referees receive. Samuel (2017) pointed out on the increasing level of professionalism and on the structured training programs for Israeli referees. The Israeli Basketball Referees Union indeed works hard to train its referees, and has an organizational culture that promotes accurate and unbiased refereeing where the quality of referees’ nominations most often depend on their performance. Deutscher (2015) argues that if the organization punishes biased decision making, referees have an extra incentive for impartial behavior, whereas organizational culture is known to be an important factor in determining employees’ performance (Ogbonna and Harris, 2000; Hogan and Coote, 2014).

The Israeli Basketball Referees Union is presently managed (at the time of our study) by two former elite-level international referees (e.g., officials at Olympic Games’ finals), who currently serve as commissioners and referees’ instructors for FIBA (the International Basketball Federation). They guide Israeli referees to avoid foul calls for marginal contact (regardless of the players involved) and to maintain consistency in their decisions. Experience, evaluation, and feedback are known to be important factors in learning and improvement in decision making (Pizzera and Raab, 2012; Renden et al., 2014; Erev and Haruvy, 2016). Guidance of Israeli referees involves feedback to the individuals or teams of referees, with weekly video materials and letters being distributed after each round of games. This is consistent with findings on the ability to improve officials’ decision making by implementing video-based training routines (Mascarenhas et al., 2005; Schweizer et al., 2011; Put et al., 2013; Put et al., 2016).

In addition, The Israeli Basketball Referees Union could fear “bad press” in case biased refereeing becomes publically known. In this respect, Deutscher (2015) has already proposed that fear of “bad press” is a possible mitigating factor. The lessons learned from the literature suggest that awareness and knowledge of bias may lead to behavioral changes (e.g., Pope et al., 2018), and therefore, over the years, a growing awareness of biases among referees could have alleviated favoritism. In our inquiry, since a number of biases were combined and studied, it was possible to adopt a context-specific approach, and to examine carefully the influence of these biases on processes of decision-making.

## Author Contributions

EM, OA, RL, ES and MB-E devised the project and interpreted the results. EM and OA performed the data analysis. EM collected the data and drafted the manuscript. OA performed critical revision of the manuscript.

## Conflict of Interest Statement

The authors declare that the research was conducted in the absence of any commercial or financial relationships that could be construed as a potential conflict of interest.

## References

[B1] Anderson K. J. Pierce D. A. (2009). Officiating bias: the effect of foul differential on foul calls in NCAA basketball. *J. Sports Sci.* 27 687–694. 10.1080/02640410902729733 19424898

[B2] Balmer N. J. Nevill A. M. Lane A. M. (2005). Do judges enhance home advantage in European championship boxing? *J. Sports Sci.* 23 409–416. 10.1080/02640410400021583 16089185

[B3] Brand R. Schmidt G. Schneeloch Y. (2006). Sequential effects in elite basketball referees’ foul decisions: an experimental study on the concept of game management. *J. Sport Exerc. Psychol.* 28 93–99. 10.1123/jsep.28.1.93

[B4] Caudill S. B. Mixon F. G. Jr. Wallace S. (2014). Life on the red carpet: star players and referee bias in the National Basketball Association. *Int. J. Econ. Bus.* 21 245–253. 10.1080/13571516.2014.903110

[B5] Dawson P. M. (2012). Experience, social pressure and performance: the case of soccer officials. *Appl. Econ. Lett.* 19 883–886. 10.1080/13504851.2011.607118

[B6] Dawson P. M. Dobson S. (2010). The influence of social pressure and nationality on individual decisions: evidence from the behaviour of referees. *J. Econ. Psychol.* 31 181–191. 10.1016/j.joep.2009.06.001

[B7] Deutscher C. (2015). No referee bias in the NBA: new evidence with leagues’ assessment data. *J. Sports Anal.* 1 91–96. 10.3233/JSA-150012

[B8] Dohmen T. Sauermann J. (2016). Referee bias. *J. Econ. Surv.* 30 679–695. 10.1111/joes.12106

[B9] Erev I. Haruvy E. (2016). “Learning and the economics of small decisions,” in *The Handbook of Experimental Economics*, Vol. 2, eds Kagel J. H. Roth A. E. (Princeton, NJ: Princeton University Press.

[B10] Findlay L. C. Ste-Marie D. M. (2004). A reputation bias in figure skating judging. *J. Sport Exerc. Psychol.* 26 154–166. 10.1123/jsep.26.1.154

[B11] Garicano L. Palacios-Huerta I. Prendergast C. (2005). Favoritism under social pressure. *Rev. Econ. Stat.* 87 208–216. 10.1162/0034653053970267

[B12] Gift P. (2017). Performance evaluation and favoritism: evidence from mixed martial arts. *J. Sports Econ.* 19 1147–1173. 10.1177/1527002517702422

[B13] Gift P. Rodenberg R. M. (2014). Napoleon complex: height bias among national basketball association referees. *J. Sports Econ.* 15 541–558. 10.1177/1527002514535168

[B14] Hogan S. J. Coote L. V. (2014). Organizational culture, innovation, and performance: a test of Schein’s model. *J. Bus. Res.* 67 1609–1621. 10.1016/j.jbusres.2013.09.007

[B15] Jones M. V. Paull G. C. Erskine J. (2002). The impact of a team’s aggressive reputation on the decisions of association football referees. *J. Sports Sci.* 20 991–1000. 10.1080/026404102321011751 12477009

[B16] Lago-Peñas C. Gómez-López M. (2016). The influence of referee bias on extra time in elite football matches. *Percept. Mot. Skills* 122 666–677. 10.1177/0031512516633342 27166341

[B17] Lehman D. R. Reifman A. (1987). Spectator influence on basketball officiating. *J. Soc. Psychol.* 127 673–675.

[B18] Mascarenhas D. R. D. Collins D. Mortimer P. Morris R. L. (2005). A naturalistic approach to training accurate and coherent decision making in rugby union referees. *Sport Psychol.* 19 131–147. 10.1123/tsp.19.2.131

[B19] Mills B. M. (2014). Social pressure at the plate: inequality aversion, status, and mere exposure. *Manag. Decis. Econ.* 35 387–403. 10.1002/mde.2630

[B20] Morgulev E. Azar O. Lidor R. Sabag E. Bar-Eli M. (2014). Deception and decision making in professional basketball: Is it beneficial to flop? *J. Econ. Behav. Org.* 102 108–118. 10.1016/j.jebo.2014.03.022

[B21] Myers T. Nevill A. Al-Nakeeb Y. (2012). The influence of crowd noise upon judging decisions in Muay Thai. *Adv. Phys. Educ.* 2 148–152. 10.4236/ape.2012.24026

[B22] Nevill A. M. Balmer N. J. Williams A. M. (2002). The influence of crowd noise and experience upon refereeing decisions in football. *Psychol. Sport Exerc.* 3 261–271. 10.1016/S1469-0292(01)00033-4

[B23] Ogbonna E. Harris L. C. (2000). Leadership style, organizational culture and performance: empirical evidence from UK companies. *Int. J. Hum. Res. Manag.* 11 766–788. 10.1080/09585190050075114

[B24] Page K. Page L. (2010). Alone against the crowd: individual differences in referees’ ability to cope under pressure. *J. Econ. Psychol.* 31 192–199. 10.1016/j.joep.2009.08.007

[B25] Pizzera A. Raab M. (2012). Perceptual judgments of sports officials are influenced by their motor and visual experience. *J. Appl. Sport Psychol.* 24 59–72. 10.1080/10413200.2011.608412

[B26] Pope D. G. Price J. Wolfers J. (2018). Awareness reduces racial bias. *Manag. Sci.* 64 4988–4995. 10.1287/mnsc.2017.2901

[B27] Price J. Remer M. Stone D. F. (2012). Subperfect game: profitable biases of NBA referees. *J. Econ. Manag. Strat.* 21 271–300. 10.1111/j.1530-9134.2011.00325.x

[B28] Put K. Wagemans J. Jaspers A. Helsen W. F. (2013). Web-based training improves on-field offside decision-making performance. *Psychol. Sport Exerc.* 14 577–585. 10.1016/j.psychsport.2013.03.005

[B29] Put K. Wagemans J. Spitz J. Williams A. M. Helsen W. F. (2016). Using web-based training to enhance perceptual-cognitive skills in complex dynamic offside events. *J. Sport Sci.* 34 181–189. 10.1080/02640414.2015.1045926 25972094

[B30] Renden P. G. Kerstens S. Oudejans R. R. Cañal-Bruland R. (2014). Foul or dive? Motor contributions to judging ambiguous foul situations in football. *Eur. J. Sport Sci.* 14(suppl. 1), S221–S227. 10.1080/17461391.2012.683813 24444210

[B31] Samuel R. D. (2017). Training prospective soccer referees using a deliberate practice perspective: The Israeli Excellence Program. *J. Sport Psychol. Action* 8 184–196. 10.1080/21520704.2017.1287798

[B32] Schweizer G. Plessner H. Kahlert D. Brand R. (2011). A video-based training method for improving soccer referees’ intuitive decision-making skills. *J. Appl. Sport Psychol.* 23 429–442.? 10.1080/10413200.2011.555346

[B33] Strauss B. Pier M. (2002). Urteilsverzerrungen von Fußballschiedsrichtern. in *Proceedings of the Vortrag auf dem 43. Kongress der Deutschen Gesellschaft für Psychologie*, Berlin.

[B34] Sutter M. Kocher M. G. (2004). Favoritism of agents–the case of referees’ home bias. *J. Econ. Psychol.* 25 461–469. 10.1016/S0167-4870(03)00013-8 29456496

[B35] Unkelbach C. Memmert D. (2010). Crowd noise as a cue in referee decisions contributes to the home advantage. *J. Sport Exerc. Psychol.* 32 483–498. 10.1123/jsep.32.4.483 20733209

[B36] Van Quaquebeke N. Giessner S. R. (2010). How embodied cognitions affect judgments: height-related attribution bias in football foul calls. *J. Sport Exerc. Psychol.* 32 3–22. 10.1123/jsep.32.1.3 20167949

